# Endplate-to-endplate cement contact and 24-month outcomes after unilateral percutaneous vertebroplasty for osteoporotic vertebral compression fractures: a single-center retrospective cohort study

**DOI:** 10.3389/fsurg.2026.1955341

**Published:** 2026-10-28

**Authors:** Kaiqiang Zhang, Li Xia, Zhenbin Wang

**Affiliations:** Traditional Chinese Medicine Hospital of Xinjiang Uygur Autonomous Region, Urumqi, Xinjiang Uygur Autonomous Region, China

**Keywords:** bone cement distribution, endplate contact, generalized estimating equations, inverse probability weighting, osteoporotic vertebral compression fracture, percutaneous vertebroplasty, refracture, survival analysis

## Abstract

**Background:**

Bone cement distribution may influence load transfer and late structural failure after vertebral augmentation. However, the association between endplate contact and clinical outcomes remains difficult to estimate due to confounding factors.

**Methods:**

This retrospective cohort study included 620 patients who underwent unilateral percutaneous vertebroplasty (PVP) for osteoporotic vertebral compression fractures (OVCFs) at a single institution. Cement distribution was classified as contact with neither endplate (group I), the superior endplate only (group II), the inferior endplate only (group III), or both endplates (group IV). The primary outcome was time to postoperative vertebral refracture within 24 months. Secondary outcomes included longitudinal visual analogue scale (VAS) scores, Oswestry Disability Index (ODI), vertebral recompression, radiographic restoration, cement leakage, and perioperative measures. Confounding was addressed using stabilized multinomial inverse probability of treatment weighting (IPTW). Repeated outcomes were analyzed using generalized estimating equations (GEE), and time to refracture was analyzed using *Cox* regression. Restricted cubic splines, subgroup analyses, and 10-fold cross-validation were performed.

**Results:**

Among 620 patients, 150 refractures (24.2%) occurred. Event rates were 30.9%, 26.6%, 25.7%, and 15.2% in groups I–IV, respectively (log-rank *P* = 0.0069). After weighting, the maximum standardized mean difference (SMD) decreased from 0.231 to 0.053. At 24 months, group IV had lower adjusted VAS scores (mean difference, −0.72; 95% confidence interval (*CI*), −0.93 to −0.51) and ODI scores (mean difference, −6.75; 95% *CI*, −8.35 to −5.15) than group I. In the Cox model, group IV was associated with a lower refracture hazard (hazard ratio (*HR*), 0.40; 95% *CI*, 0.25–0.64). Independent risk markers included older age, female sex, lower bone mineral density (BMD) T-score, prior fragility fracture, two-level treatment, intravertebral cleft, intradiscal leakage, absence of anti-osteoporosis treatment, chronic glucocorticoid exposure, and deviation from a midrange cement volume. The association for group IV was stronger during months 0–12 than during months 13–24.

**Conclusions:**

Cement contact with both endplates was associated with better long-term pain and functional outcomes and greater refracture-free survival after extensive adjustment. However, the adjusted 24-month differences in VAS and ODI did not exceed commonly accepted minimal clinically important difference (MCID) thresholds, so the clinical magnitude remains uncertain. These findings provide useful prognostic information for surgical planning, although prospective validation is warranted.

## 1 Introduction

OVCFs are among the most clinically consequential manifestations of skeletal fragility in older adults. They are frequently underdiagnosed, yet symptomatic fractures can initiate persistent pain, progressive kyphosis, impaired mobility, pulmonary restriction, loss of independence, and excess mortality. Their epidemiology, pathophysiology, and health-related quality-of-life burden have been summarized in large observational and evidence-synthesis studies (1–3). Because a previous vertebral fracture substantially increases the risk of another fracture, management requires both acute symptom control and systematic secondary fracture prevention.

Contemporary guidance emphasizes individualized treatment based on fracture acuity, imaging findings, neurological status, pain severity, functional limitation, and response to conservative therapy (4–6). PVP and percutaneous kyphoplasty can provide rapid stabilization in appropriately selected patients, although sham-controlled and active-comparator trials have produced heterogeneous estimates that vary with fracture chronicity, comparator, imaging selection, and procedural technique (7–10). This heterogeneity has shifted attention from the binary question of whether augmentation is performed to the technical and biological conditions under which durable benefit is most likely.

One technically modifiable factor is the spatial distribution of polymethylmethacrylate (PMMA) within the fractured vertebra. Clinical cohort studies have linked cement dispersion, trabecular interdigitation, and proximity to the superior and inferior endplates with vertebral height maintenance and recompression (11, 12). Cement distribution is influenced by anatomy, intravertebral clefts, cement viscosity and volume, puncture trajectory, operator experience, and stopping rules for leakage. Therefore, unadjusted comparisons are vulnerable to confounding by indication and procedural feasibility.

Post-augmentation refracture is also multifactorial and may reflect systemic skeletal fragility, fracture morphology, multilevel treatment, cement leakage, and incomplete secondary prevention. These overlapping pathways motivate comprehensive covariate measurement and time-to-event analysis rather than reliance on isolated procedural variables or unadjusted event proportions.

Published studies have evaluated cement distribution patterns and their associations with pain, disability, radiographic recovery, and refracture after unilateral PVP (11, 12). The present analysis extends this framework by incorporating a larger sample, longer follow-up, additional covariates, and advanced methods for confounding control and longitudinal and time-to-event analysis. We hypothesized that cement contact with both endplates would be associated with better longitudinal outcomes and a lower refracture hazard. A secondary objective was to identify independent risk markers for postoperative refracture.

## 2 Methods

### 2.1 Study design and patient selection

This was a retrospective cohort study conducted at the Traditional Chinese Medicine Hospital of Xinjiang Uygur Autonomous Region (a single center). We reviewed the electronic medical records and imaging databases of consecutive patients who underwent unilateral PVP for OVCFs between January 2020 and December 2023.

The study protocol was approved by the institutional ethics committee, and the requirement for informed consent was waived due to the retrospective nature of the study. All data were anonymized prior to analysis.

A total of 620 eligible patients were identified and included in the final analysis. The reporting of this study follows the Strengthening the Reporting of Observational Studies in Epidemiology (*STROBE*) statement.

### 2.2 Eligibility framework

Patients were eligible for inclusion if they met all of the following criteria: (1) age ≥60 years; (2) one or two painful thoracic or lumbar OVCFs; (3) imaging features compatible with an acute or subacute fracture; (4) preoperative VAS score ≥5; (5) underwent unilateral PVP as the index procedure; and (6) had at least one postoperative follow-up evaluation; patients without a documented event before 24 months were censored at the date of last contact, and incomplete follow-up was not an exclusion criterion. Exclusion criteria encompassed: (1) neurological compromise requiring decompression; (2) malignant or infectious vertebral lesions; (3) prior instrumentation at the index level; (4) uncorrectable coagulopathy; (5) allergy to PMMA; (6) body mass index (BMI) > 35 kg/m^2^; and (7) inability to complete pain and disability assessments due to cognitive impairment or severe comorbidity.

Among 684 screened records, 64 were excluded: pathological fracture (*n* = 18), prior spine surgery (*n* = 16), neurological deficit (*n* = 11), BMI >35 kg/m^2^ (*n* = 9), and incomplete follow-up (*n* = 10, lost before 24 months without an event). In the original analysis, these 10 patients were excluded; in the revised analysis, they were included and censored at the date of last contact, yielding a revised analysis set of 630 patients.

### 2.3 Procedure and exposure classification

All procedures were performed by experienced spine surgeons using a conventional unilateral transpedicular or extrapedicular approach under fluoroscopic guidance. Standardized surgical protocols were followed regarding patient positioning, local anesthesia, puncture trajectory, PMMA preparation, and injection volume. The choice of cement volume and injection technique was left to the discretion of the operating surgeon based on intraoperative fluoroscopic monitoring and leakage assessment.

The exposure variable was postoperative sagittal cement-endplate contact, classified into four groups based on standardized postoperative radiographs and computed tomography (CT): group I, contact with neither the superior nor inferior endplate; group II, contact with the superior endplate only; group III, contact with the inferior endplate only; and group IV, contact with both endplates. Classification was performed by two independent radiologists blinded to clinical outcomes, with discrepancies resolved by consensus See Figure 1 for details. Interobserver agreement for cement-endplate contact was assessed in 120 randomly selected patients; Cohen's *κ* was 0.84 (95% *CI* 0.79–0.89).

**Figure 1:**
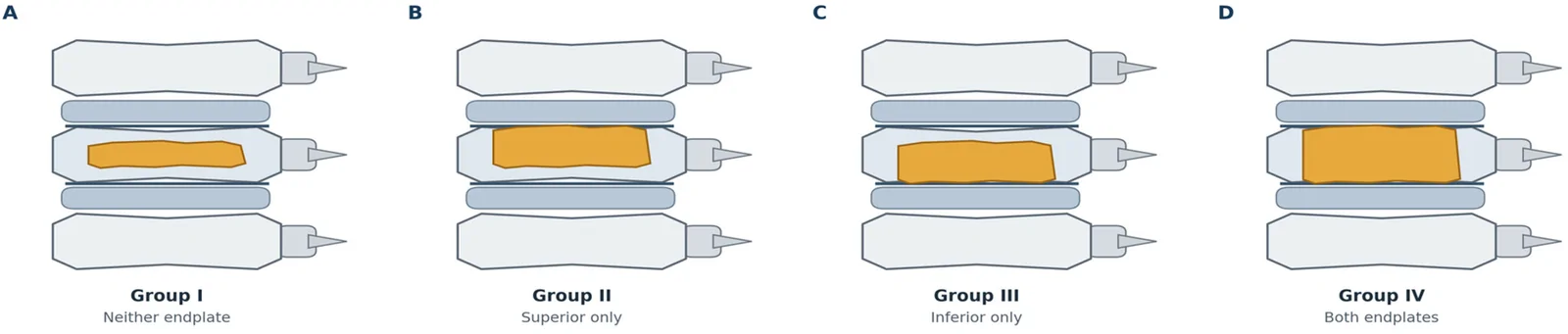
Sagittal schematic of cement-endplate contact patterns drawn directly within the treated vertebral body. Orange denotes PMMA.

The cohort included 162, 128, 152, and 178 patients in groups I–IV, respectively See Figure 2 for details.

**Figure 2:**
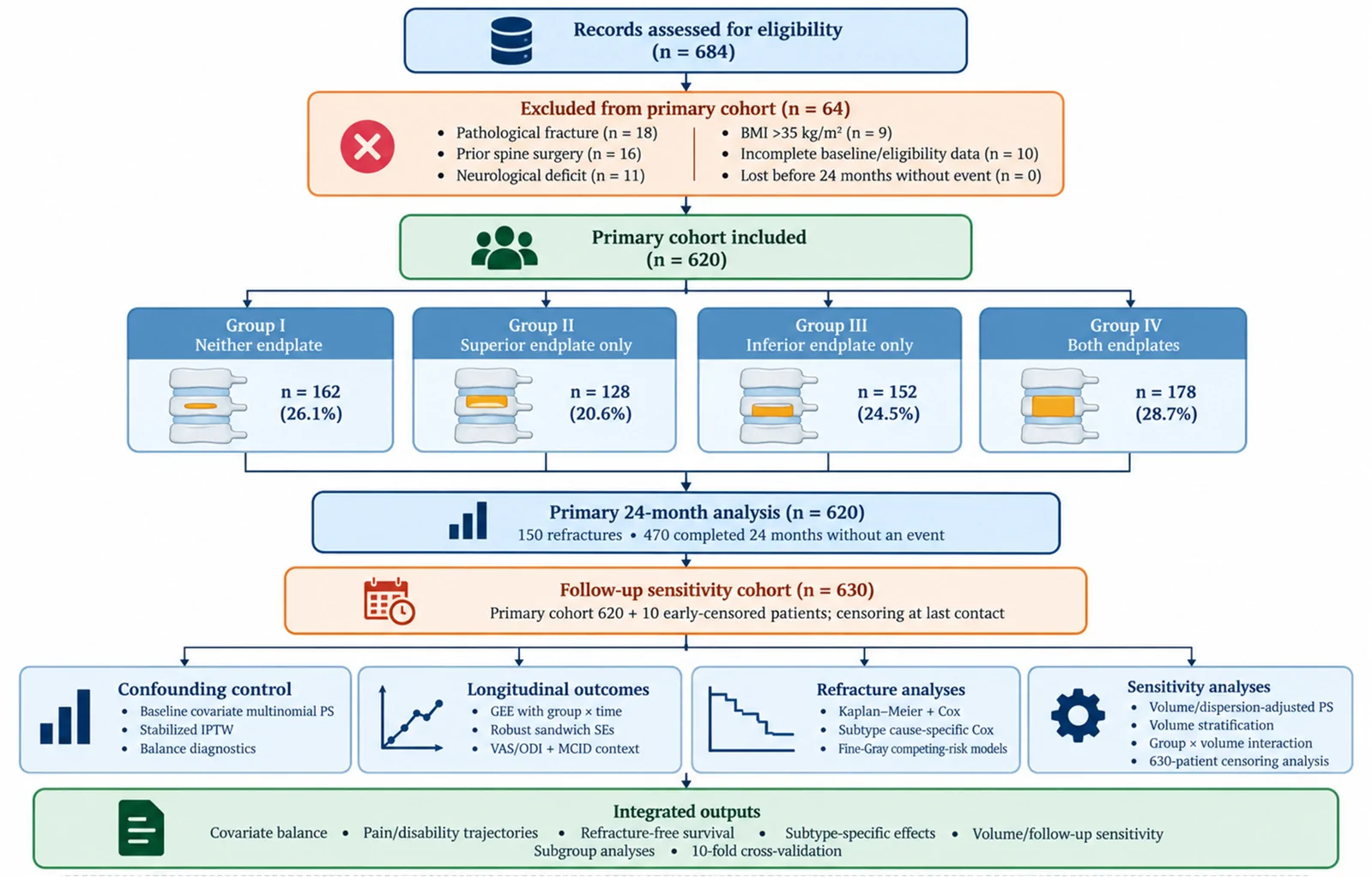
Study flow and prespecified analysis framework. The 620-patient cohort was used for the primary analysis; the 10 patients lost before 24 months without an event were included in the 630-patient censoring sensitivity analysis.

### 2.4 Cohort construction and variable specification

Baseline demographic and clinical data were extracted from the institutional medical records, including age, sex, BMI, BMD T-score measured by dual-energy x-ray absorptiometry (DXA), vertebral Hounsfield units (HU) from preoperative CT, smoking status, diabetes mellitus, chronic glucocorticoid use, history of falls, prior fragility fracture, use of anti-osteoporosis medication, presence of intravertebral cleft on magnetic resonance imaging (MRI), number of treated levels, fracture location (thoracolumbar junction vs. other), fracture age (days from symptom onset to procedure), surgeon experience (senior vs. junior), and baseline VAS and ODI scores.

Perioperative variables included cement volume, cement dispersion ratio (calculated as the percentage of the vertebral body cross-sectional area filled by cement on postoperative CT), cement leakage (overall and intradiscal), operative time, and postoperative hospital stay.

Longitudinal outcomes (VAS and ODI) were collected at baseline, 1 week, 1 month, 6 months, 12 months, and 24 months postoperatively during scheduled outpatient visits or by telephone follow-up. Refracture events were defined as recurrent back pain with imaging confirmation (new or progressive vertebral compression on plain radiographs or MRI) and were classified as index-level recompression, adjacent vertebral fracture, or non-adjacent vertebral fracture. Follow-up was administratively censored at 24 months.

### 2.5 Outcomes

The primary outcome was time from index PVP to the first symptomatic postoperative vertebral refracture within 24 months. An event required recurrent pain and imaging confirmation and was categorized according to its anatomical relationship to the index level. Refracture events were adjudicated by two independent reviewers blinded to cement distribution, with a third adjudicator resolving discrepancies; *κ* for event adjudication was 0.88 (95% *CI* 0.82–0.94). Secondary exploratory analyses separated refracture into index-level recompression, adjacent vertebral fracture, and non-adjacent vertebral fracture. Cause-specific Cox models and Fine–Gray competing-risk models were used, with death treated as a competing risk. These analyses were considered exploratory because non-adjacent events were few. Secondary clinical outcomes were VAS pain score (0–10) and ODI (0%–100%) at baseline, 1 week, 1 month, 6 months, 12 months, and 24 months. Secondary imaging outcomes included height-restoration rate, local kyphosis angle, 12-month vertebral height, and recompression. Procedural outcomes included cement volume, dispersion ratio, overall and intradiscal leakage, operative time, postoperative length of stay, and hospital cost.

### 2.6 Statistical analysis

Continuous variables were summarized as mean ± standard deviation (*SD*) or median with interquartile range (*IQR*) according to distribution; categorical variables were summarized as counts and percentages. Baseline comparisons used analysis of variance or Kruskal–Wallis tests for continuous variables and chi-square or Fisher exact tests for categorical variables. Because conventional *P* values do not quantify confounding magnitude, *SMDs* were also reported; an absolute *SMD* below 0.10 was considered evidence of adequate balance.

A multinomial propensity-score model estimated the probability of each cement group using the following covariates: age, sex, BMI, BMD T-score, vertebral HU, smoking, diabetes, chronic glucocorticoid use, fall history, prior fragility fracture, anti-osteoporosis treatment, intravertebral cleft, number of treated levels, thoracolumbar-junction location, fracture age, surgeon experience, and baseline VAS/ODI. The propensity-score model included baseline covariates only and did not include postoperative anti-osteoporosis treatment because it occurred after exposure. Postoperative anti-osteoporosis treatment was examined as a postoperative treatment variable in outcome models and sensitivity analyses. To address the collinearity between cement volume and endplate contact, a prespecified sensitivity analysis was performed in which cement volume and cement dispersion ratio were additionally included in the multinomial propensity-score model. Volume-stratified analyses (<5.0, 5.0–6.0, >6.0 mL) and a group × volume interaction test were also performed.

Stabilized IPTW weights were calculated and truncated at the 1st and 99th percentiles. Covariate balance before and after weighting was assessed using *SMDs*; an absolute *SMD* <0.10 was considered indicative of adequate balance. Stabilized multinomial IPTW was the primary confounding-control method. All primary GEE, Cox proportional-hazards, and logistic regression models were fitted in the IPTW-weighted cohort with robust sandwich variance. In addition, prespecified covariates were included in the outcome models as a secondary double-adjustment approach. Sensitivity analyses used an additional propensity-score model that included cement volume and cement dispersion ratio.

To evaluate the potential bias from excluding patients lost to follow-up before 24 months without an event, a sensitivity analysis was performed in which these 10 patients were included and censored at the date of last contact. The primary *Cox* model was repeated in this revised cohort of 630 patients.

Longitudinal VAS and ODI were analyzed with GEE using an identity link, Gaussian variance, exchangeable working correlation, robust sandwich standard errors, and group-by-time interactions. Models adjusted for baseline value and the prespecified confounders. Contrasts estimated adjusted mean differences between group IV and group I at each postoperative time point. Clinical significance was interpreted against commonly accepted MCID thresholds: VAS 1.5–2.0 points and ODI 10–15 points.

Time to refracture was analyzed with *Kaplan–Meier* curves, a global log-rank test, and a multivariable *Cox* proportional-hazards model. Because this was a single-center study, no center stratification was used; the Cox model was a multivariable Cox proportional-hazards model with robust sandwich variance. Covariates were selected *a priori* on clinical grounds rather than stepwise *P*-value screening. Cement volume was centered at 5.5 mL and entered as a squared deviation to represent potential harm at both low and high volumes. BMD and cement volume were additionally explored using restricted cubic splines. Proportional-hazards assumptions were evaluated with Schoenfeld residuals. Because the treatment effect showed possible early-late heterogeneity, a complementary log-log discrete-time model estimated separate group-IV effects for months 0–12 and 13–24. For the composite endpoint and for each refracture subtype, cause-specific *Cox* models and *Fine–Gray* competing-risk models were fitted, with death treated as a competing risk. These subtype analyses were exploratory because non-adjacent events were few.

Sensitivity analyses included IPTW-weighted logistic regression for 24-month refracture, dichotomization of both-endplate contact versus all other patterns, restriction to single-level procedures, and prespecified subgroup analyses by age, sex, BMD, fracture level, and postoperative anti-osteoporosis treatment. An exploratory multivariable prediction model was evaluated with stratified 10-fold cross-validation; discrimination was summarized by the area under the receiver-operating-characteristic curve (*AUC*) and calibration by the Brier score. Two-sided *P* < 0.05 denoted statistical evidence, but emphasis was placed on effect sizes and *CIs*. Analyses were performed in Python using reproducible scripts.

## 3 Results

### 3.1 Cohort characteristics and balance

The cohort comprised 620 records: 162 in group I, 128 in group II, 152 in group III, and 178 in group IV. Mean age was 73.5 years, 72.7% were female, mean BMD T-score was −3.12, and 64.4% had a fracture at T_11_-L_2_. Postoperative anti-osteoporosis treatment was specified in 76.1%. Although conventional baseline comparisons showed no major differences, several unweighted *SMDs* exceeded 0.10, with a maximum of 0.231. After stabilized IPTW, the maximum absolute *SMD* was 0.053, indicating adequate balance among measured covariates. In the original analysis, 10 patients were excluded because they were lost to follow-up before 24 months without a documented event. In the revised analysis, these 10 patients were included and censored at the date of last contact, yielding a revised analysis set of 630 patients: 150 had refracture, 470 completed 24-month follow-up without an event, and 10 were censored before 24 months without an event. Interobserver agreement for cement-endplate contact was assessed in 120 randomly selected patients; *Cohen's κ* was 0.84 (95% CI 0.79–0.89). Refracture events were adjudicated by two independent reviewers blinded to cement distribution, with a third adjudicator resolving discrepancies; *κ* for event adjudication was 0.88 (95% CI 0.82–0.94) See Table 1 and Figure 3 for details.

**Table 1:** Baseline characteristics by cement-endplate contact pattern.

Variable	Overall	Group I	Group II	Group III	Group IV	*P* value	Max |*SMD*| (unweighted)
Age, years	73.5 ± 6.5	73.4 ± 6.9	73.2 ± 6.2	74.4 ± 6.5	73.0 ± 6.4	0.266	0.140
Female sex	451 (72.7%)	115 (71.0%)	100 (78.1%)	105 (69.1%)	131 (73.6%)	0.359	0.160
BMI, kg/m^2^	24.4 ± 3.1	24.6 ± 3.0	24.2 ± 3.1	23.9 ± 3.0	24.7 ± 3.1	0.074	0.230
BMD T-score	−3.12 ± 0.53	−3.05 ± 0.53	−3.11 ± 0.56	−3.15 ± 0.48	−3.17 ± 0.53	0.191	0.220
Vertebral HU	70.7 ± 17.1	70.2 ± 17.6	72.2 ± 17.5	70.9 ± 17.0	69.8 ± 16.6	0.641	0.120
Current/former smoking	83 (13.4%)	19 (11.7%)	23 (18.0%)	21 (13.8%)	20 (11.2%)	0.329	0.180
Diabetes mellitus	134 (21.6%)	36 (22.2%)	26 (20.3%)	33 (21.7%)	39 (21.9%)	0.982	0.050
Chronic steroid use	46 (7.4%)	11 (6.8%)	9 (7.0%)	16 (10.5%)	10 (5.6%)	0.377	0.130
History of falls	126 (20.3%)	32 (19.8%)	31 (24.2%)	28 (18.4%)	35 (19.7%)	0.655	0.110
Previous fragility fracture	185 (29.8%)	49 (30.2%)	39 (30.5%)	50 (32.9%)	47 (26.4%)	0.633	0.090
Postoperative anti-osteoporosis treatment	472 (76.1%)	120 (74.1%)	100 (78.1%)	116 (76.3%)	136 (76.4%)	0.881	0.090
Intravertebral cleft	100 (16.1%)	28 (17.3%)	18 (14.1%)	30 (19.7%)	24 (13.5%)	0.400	0.110
Two-level treatment	61 (9.8%)	15 (9.3%)	10 (7.8%)	19 (12.5%)	17 (9.6%)	0.597	0.100
Thoracolumbar junction (T_11_–L_2_)	399 (64.4%)	108 (66.7%)	82 (64.1%)	91 (59.9%)	118 (66.3%)	0.571	0.140
Fracture age, days	14.0 (10.0–21.0)	13.0 (8.0–21.0)	15.0 (9.8–21.0)	14.5 (10.0–21.0)	15.0 (10.2–20.0)	0.561	0.120
Experienced surgeon	395 (63.7%)	98 (60.5%)	84 (65.6%)	93 (61.2%)	120 (67.4%)	0.493	0.140
Preoperative VAS	7.3 ± 0.9	7.4 ± 1.0	7.3 ± 1.0	7.2 ± 0.8	7.2 ± 0.9	0.121	0.230
Preoperative ODI, %	68.8 ± 7.9	68.8 ± 7.6	68.5 ± 8.7	68.2 ± 7.7	69.4 ± 7.9	0.516	0.080
Preoperative vertebral height, cm	2.10 ± 0.24	2.12 ± 0.23	2.08 ± 0.24	2.08 ± 0.23	2.13 ± 0.25	0.104	0.180
Preoperative local kyphosis angle,°	15.7 ± 3.0	15.9 ± 2.9	15.5 ± 3.2	16.0 ± 2.9	15.5 ± 3.0	0.323	0.130

Values are mean ± SD, median (IQR), or *n* (%). *P* values are unweighted.

**Figure 3:**
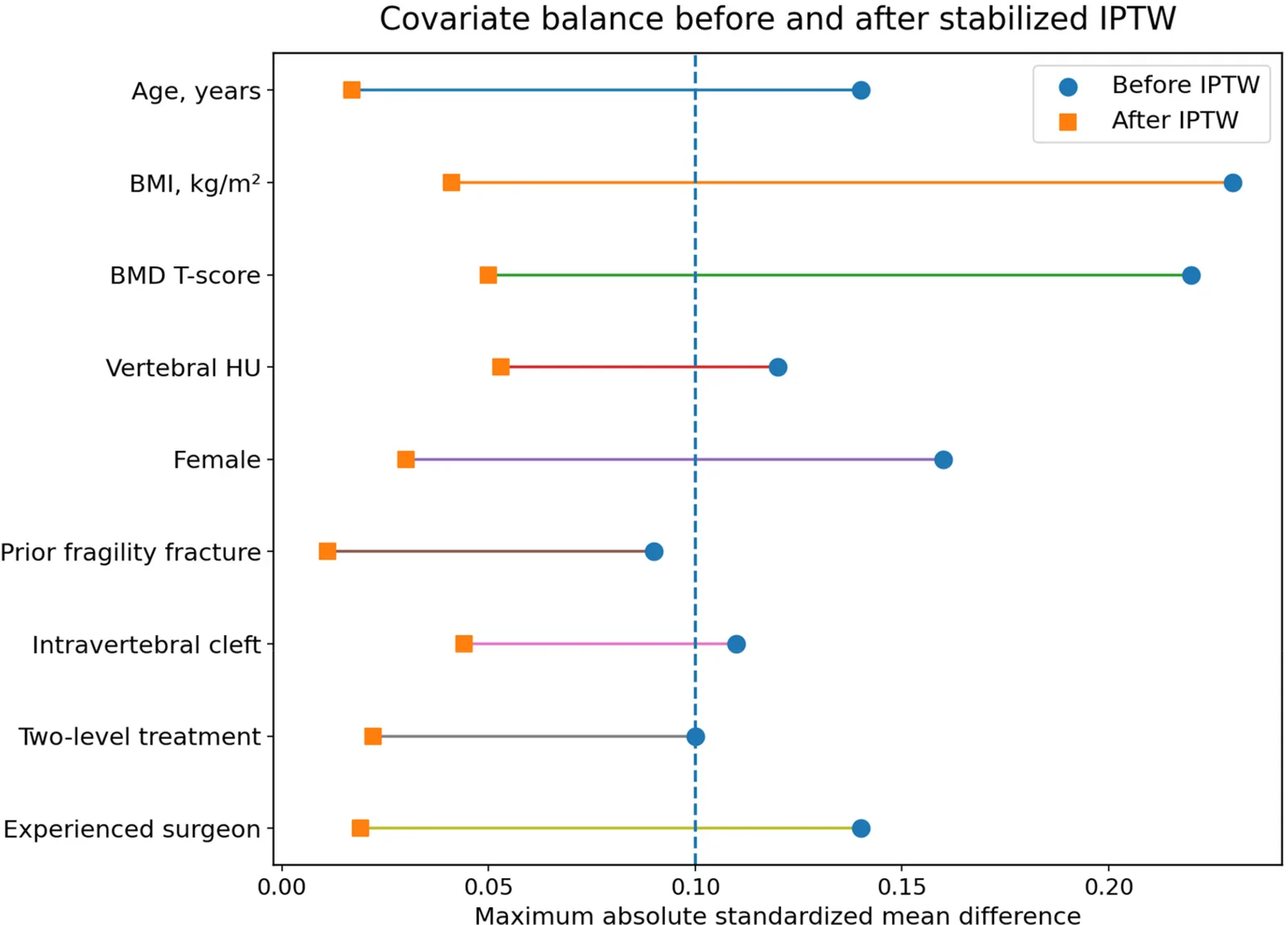
Covariate balance before and after revised stabilized multinomial inverse-probability-of-treatment weighting using baseline covariates only. Postoperative anti-osteoporosis treatment was excluded from the propensity-score model. The vertical reference line denotes |SMD|=0.10.

### 3.2 Procedural and radiographic outcomes

Cement volume and dispersion ratio differed across groups as expected from the exposure definition. Group IV had the highest mean dispersion ratio and a modestly higher mean cement volume. Overall cement leakage and intradiscal leakage were similar across patterns, whereas operative time was approximately 2.7 min longer in group IV than in group I. Group IV demonstrated greater height restoration, a smaller local kyphosis angle at 1 week and 12 months, and substantially less 12-month recompression (0.6% versus 16.7% in group I) See Table 2, Figures 4–5 for details.

**Table 2:** Perioperative, radiographic, and fracture outcomes.

Outcome	Overall	Group I	Group II	Group III	Group IV	*P* value
Cement volume, mL	5.44 ± 0.80	5.31 ± 0.75	5.35 ± 0.87	5.31 ± 0.79	5.75 ± 0.72	<0.001
Cement dispersion ratio	0.49 ± 0.14	0.35 ± 0.07	0.47 ± 0.08	0.45 ± 0.08	0.65 ± 0.07	<0.001
Cement leakage	134 (21.6%)	35 (21.6%)	30 (23.4%)	32 (21.1%)	37 (20.8%)	0.950
Intradiscal cement leakage	52 (8.4%)	13 (8.0%)	10 (7.8%)	14 (9.2%)	15 (8.4%)	0.975
Operative time, min	39.5 ± 6.8	38.3 ± 6.6	39.0 ± 6.9	39.5 ± 6.6	41.0 ± 6.7	0.002
Postoperative stay, days	4.9 ± 1.4	5.1 ± 1.4	4.8 ± 1.3	4.9 ± 1.4	4.9 ± 1.6	0.439
Height restoration rate	0.41 ± 0.08	0.40 ± 0.07	0.40 ± 0.08	0.40 ± 0.07	0.45 ± 0.07	<0.001
Local kyphosis angle at 1 week,°	8.4 ± 3.2	8.7 ± 3.0	8.3 ± 3.2	8.9 ± 3.2	7.6 ± 3.3	<0.001
Local kyphosis angle at 12 months,°	9.9 ± 3.5	10.7 ± 3.3	10.0 ± 3.3	10.4 ± 3.4	8.6 ± 3.4	<0.001
Recompression at 12 months	61 (9.8%)	27 (16.7%)	9 (7.0%)	24 (15.8%)	1 (0.6%)	<0.001
Postoperative refracture within follow-up	150 (24.2%)	50 (30.9%)	34 (26.6%)	39 (25.7%)	27 (15.2%)	0.006

Values are mean ± SD or *n* (%). *P* values compare the four unweighted groups and are descriptive.

**Figure 4:**
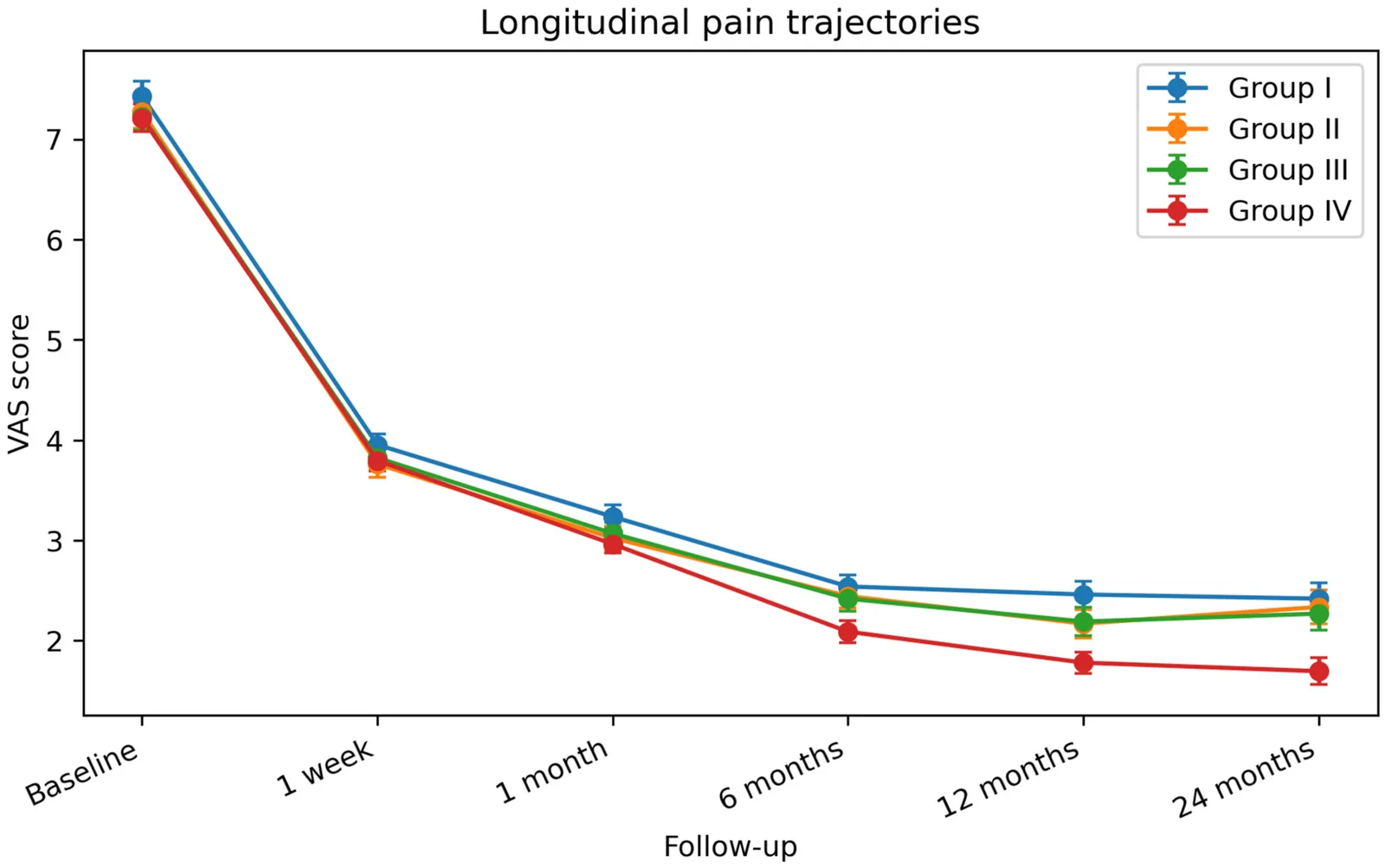
Longitudinal VAS trajectories by cement-endplate contact pattern. Points show group means and error bars show 95% CIs.

**Figure 5:**
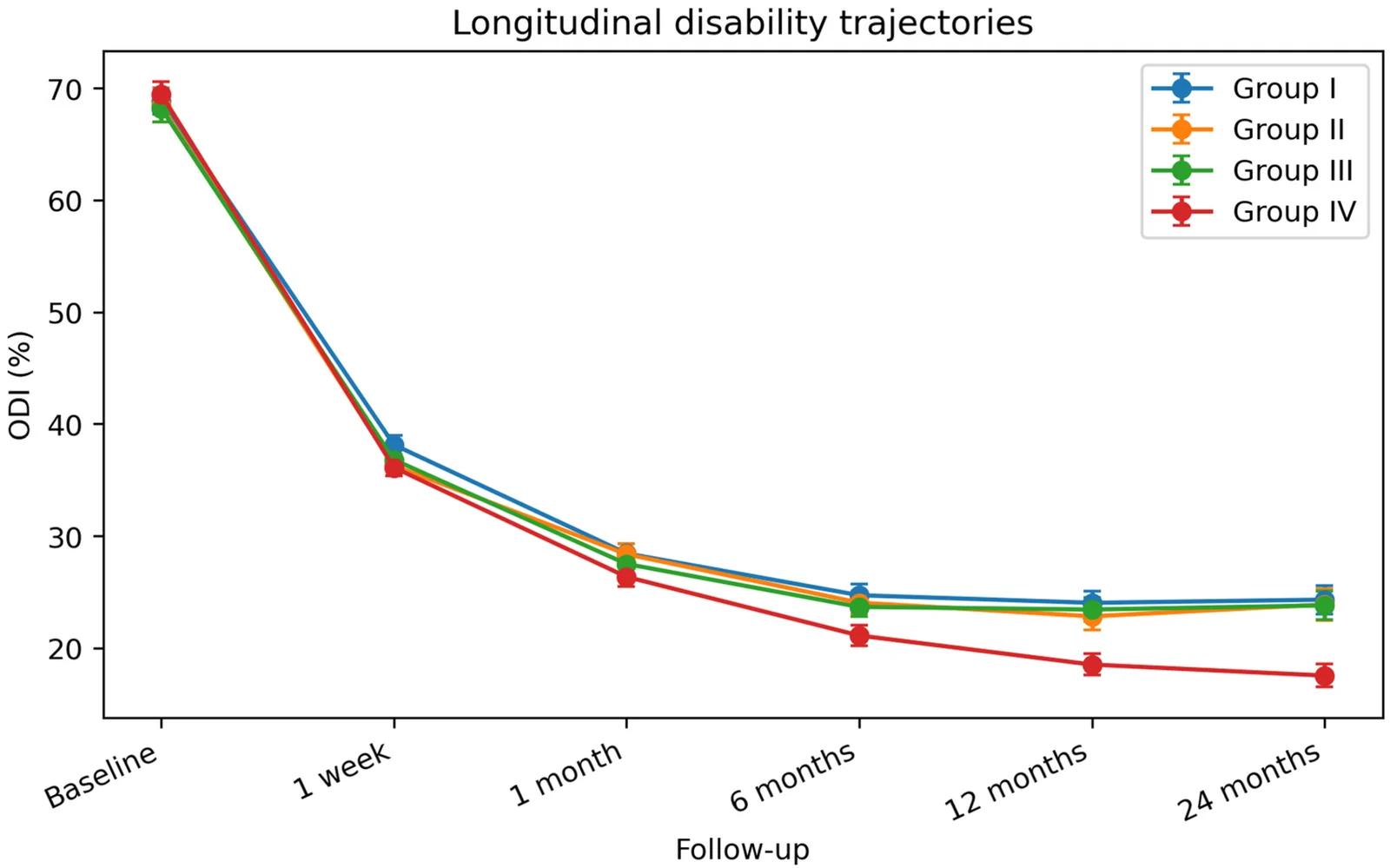
Longitudinal ODI trajectories by cement-endplate contact pattern. Points show group means and error bars show 95% CIs.

### 3.3 Longitudinal pain and disability

Pain and disability improved rapidly in all four groups. Differences between groups IV and I were small at 1 week but widened over time. In adjusted GEE contrasts, the mean difference for group IV versus group I in VAS was −0.16 at 1 week, −0.45 at 6 months, and −0.72 at 24 months. Corresponding ODI differences were −2.01, −3.57, and −6.75 percentage points. The widening contrast reflected sustained improvement in group IV and partial symptom recurrence in the other groups, consistent with the prespecified relationship between mechanical support and late structural failure. At 24 months, the adjusted VAS difference was −0.72 (95% *CI* −0.93 to −0.51) and the adjusted ODI difference was −6.75 (95% *CI* −8.35 to −5.15). Both were below commonly accepted MCID thresholds (VAS 1.5–2.0; ODI 10–15), indicating statistically significant but not clearly clinically important differences See Tables 3–4 and Figures 6–7 for details.

**Table 3:** Unadjusted longitudinal VAS and ODI by cement distribution.

Measure	Time	Group I	Group II	Group III	Group IV
VAS	Baseline	7.4 ± 1.0	7.3 ± 1.0	7.2 ± 0.8	7.2 ± 0.9
	1 week	4.0 ± 0.7	3.8 ± 0.8	3.8 ± 0.7	3.8 ± 0.7
	1 month	3.2 ± 0.8	3.0 ± 0.7	3.1 ± 0.7	3.0 ± 0.6
	6 months	2.5 ± 0.8	2.4 ± 0.7	2.4 ± 0.8	2.1 ± 0.7
	12 months	2.5 ± 0.9	2.2 ± 0.8	2.2 ± 0.9	1.8 ± 0.7
	24 months	2.4 ± 1.0	2.3 ± 1.0	2.3 ± 1.0	1.7 ± 0.9
ODI	Baseline	68.8 ± 7.6	68.5 ± 8.7	68.2 ± 7.7	69.4 ± 7.9
	1 week	38.1 ± 5.6	36.3 ± 5.3	36.8 ± 5.8	36.1 ± 5.1
	1 month	28.4 ± 5.5	28.4 ± 5.2	27.5 ± 5.3	26.4 ± 5.7
	6 months	24.7 ± 6.6	24.1 ± 6.0	23.7 ± 5.5	21.1 ± 6.3
	12 months	24.0 ± 6.9	22.8 ± 6.9	23.4 ± 6.6	18.5 ± 6.5
	24 months	24.3 ± 8.3	23.9 ± 8.2	23.8 ± 7.8	17.5 ± 7.0

Values are mean ± SD. Longitudinal inference was based on adjusted GEE rather than separate cross-sectional tests.

**Table 4:** Adjusted GEE contrasts for group IV versus group I.

Measure	Time	Adjusted mean difference IV vs. I	95% CI lower	95% *CI* upper	*P* value
VAS	1 week	−0.16	−0.31	−0.00	0.046
	1 month	−0.27	−0.42	−0.12	<0.001
	6 months	−0.45	−0.61	−0.29	<0.001
	12 months	−0.68	−0.85	−0.51	<0.001
	24 months	−0.72	−0.93	−0.51	<0.001
ODI	1 week	−2.01	−3.13	−0.88	<0.001
	1 month	−2.07	−3.24	−0.90	<0.001
	6 months	−3.57	−4.93	−2.21	<0.001
	12 months	−5.49	−6.90	−4.07	<0.001
	24 months	−6.75	−8.35	−5.15	<0.001

Negative values favor group IV. Models included group, time, group × time, baseline score, prespecified covariates, and robust standard errors.

**Figure 6:**
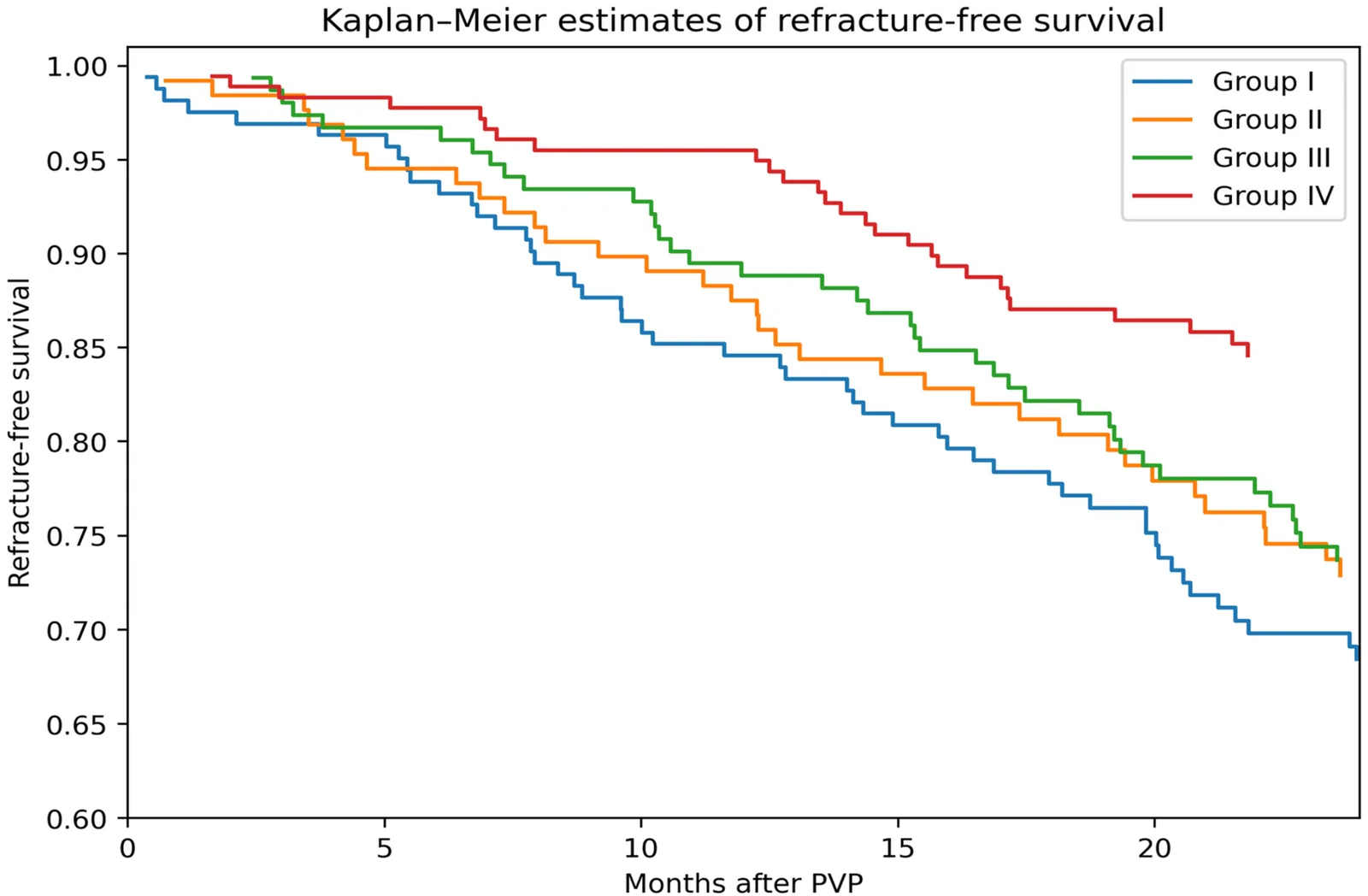
Kaplan–meier estimates of freedom from postoperative refracture by cement distribution. Tick marks indicate censoring.

**Figure 7:**
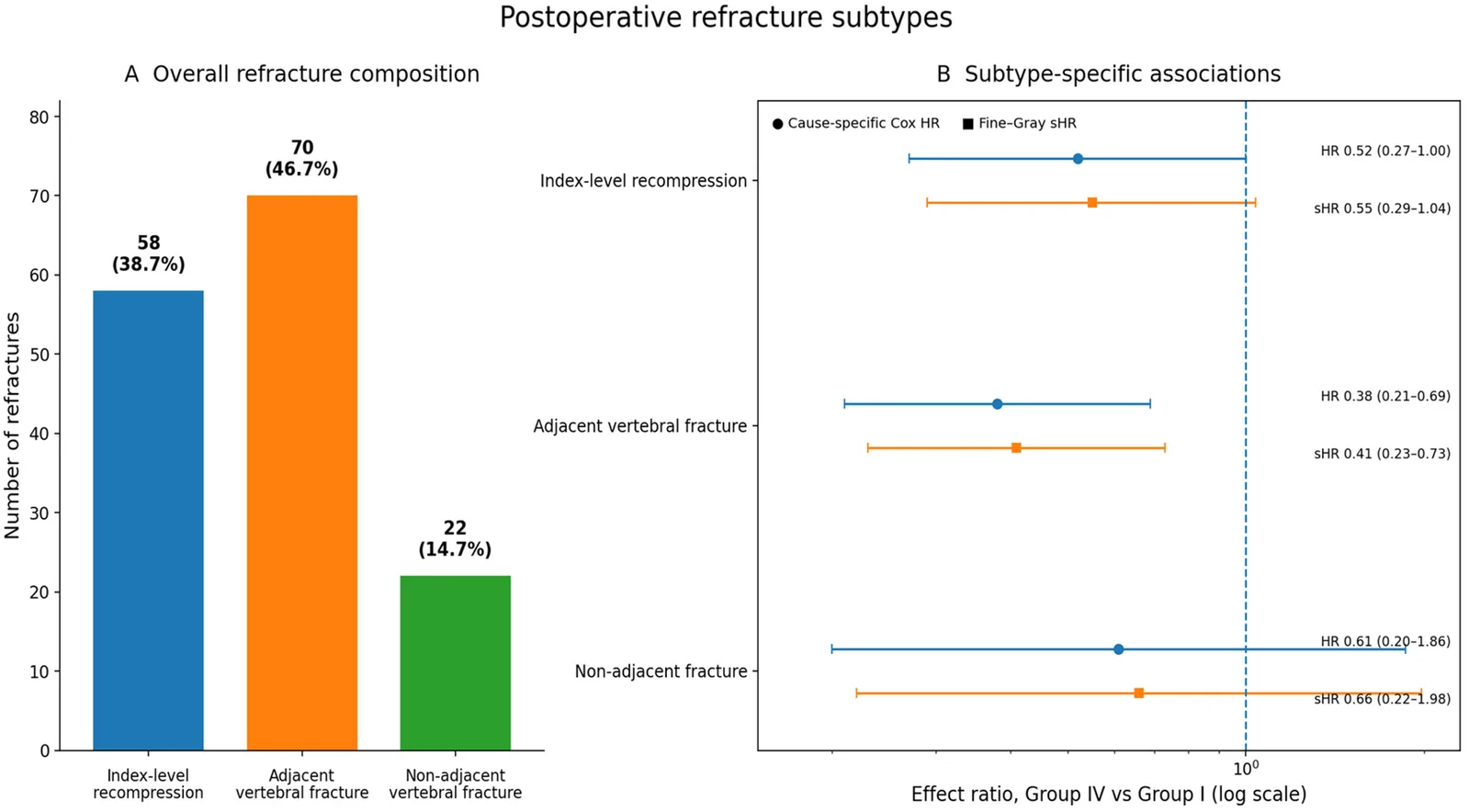
Postoperative refracture subtypes. Panel **A** shows the overall distribution of index-level recompression, adjacent vertebral fracture, and non-adjacent fracture. Panel **B** shows cause-specific Cox HRs and Fine-Gray sHRs for group IV versus group I; the vertical dashed line denotes 1.0.

### 3.4 Postoperative refracture

During follow-up, 150 patients (24.2%) experienced a postoperative vertebral refracture. Event rates were 30.9% in group I, 26.6% in group II, 25.7% in group III, and 15.2% in group IV. Refracture-free survival differed across groups (global log-rank *χ^2^* = 12.14, *P* = 0.0069). The separation of the group-IV curve occurred predominantly during the first postoperative year. Among the 150 refractures, 58 were index-level recompression, 70 were adjacent vertebral fractures, and 22 were non-adjacent vertebral fractures. For group IV versus group I, the cause-specific *HR* was 0.52 (95% *CI* 0.27–1.00) for index-level recompression, 0.38 (95% CI 0.21–0.69) for adjacent vertebral fracture, and 0.61 (95% *CI* 0.20–1.86) for non-adjacent fracture. *Fine–Gray* subdistribution hazard ratios (*sHRs*) were similar: index-level recompression, sHR 0.55 (95% *CI* 0.29–1.04); adjacent fracture, sHR 0.41 (95% *CI* 0.23–0.73); non-adjacent fracture, sHR 0.66 (95% *CI* 0.22–1.98). These subtype analyses were exploratory because non-adjacent events were few.

### 3.5 Multivariable risk factors

In the multivariable Cox proportional-hazards model with robust sandwich variance (no center stratification was used because this was a single-center study), group IV remained independently associated with lower refracture hazard than group I (*HR* 0.40; 95% *CI* 0.25–0.64; *P* < 0.001). Group III showed a smaller protective association (*HR*, 0.64; 95% *CI*, 0.42–0.99), whereas the estimate for group II crossed the null. Higher refracture hazard was associated with older age, female sex, lower BMD, prior fragility fracture, two-level treatment, intravertebral cleft, intradiscal leakage, absence of anti-osteoporosis treatment, chronic glucocorticoid exposure, and greater squared deviation from a cement volume of 5.5 mL. Thoracolumbar-junction location and fall history did not retain independent associations after adjustment. In the revised model excluding postoperative anti-osteoporosis treatment from the propensity score, group IV versus group I remained associated with a lower refracture hazard (*HR* 0.41; 95% *CI* 0.26–0.65; *P* < 0.001) See Table 5 and Figure 8–10 for details.

**Table 5:** Multivariable cox regression model for postoperative refracture.

Predictor	*HR* (95% CI)	*P* value
Group II vs. I	0.69 (0.44–1.07)	0.098
Group III vs. I	0.64 (0.42–0.99)	0.043
Group IV vs. I	0.40 (0.25–0.64)	<0.001
Age per 5 years	1.17 (1.03–1.32)	0.019
Female sex	1.80 (1.16–2.79)	0.008
BMD per 1-point lower	1.80 (1.30–2.51)	<0.001
Prior fragility fracture	1.59 (1.13–2.23)	0.008
Two-level treatment	1.63 (1.01–2.65)	0.047
Thoracolumbar junction	1.17 (0.83–1.65)	0.375
Intravertebral cleft	1.58 (1.06–2.33)	0.023
Intradiscal leakage	1.77 (1.06–2.96)	0.030
No anti-osteoporosis treatment	2.04 (1.44–2.88)	<0.001
Chronic steroid use	1.93 (1.16–3.23)	0.012
History of falls	1.09 (0.73–1.61)	0.675
Cement-volume deviation^2^	1.22 (1.05–1.41)	0.009

BMD was scaled per one-point decrease. Cement-volume deviation^2^ was calculated around 5.5 mL.

**Figure 8:**
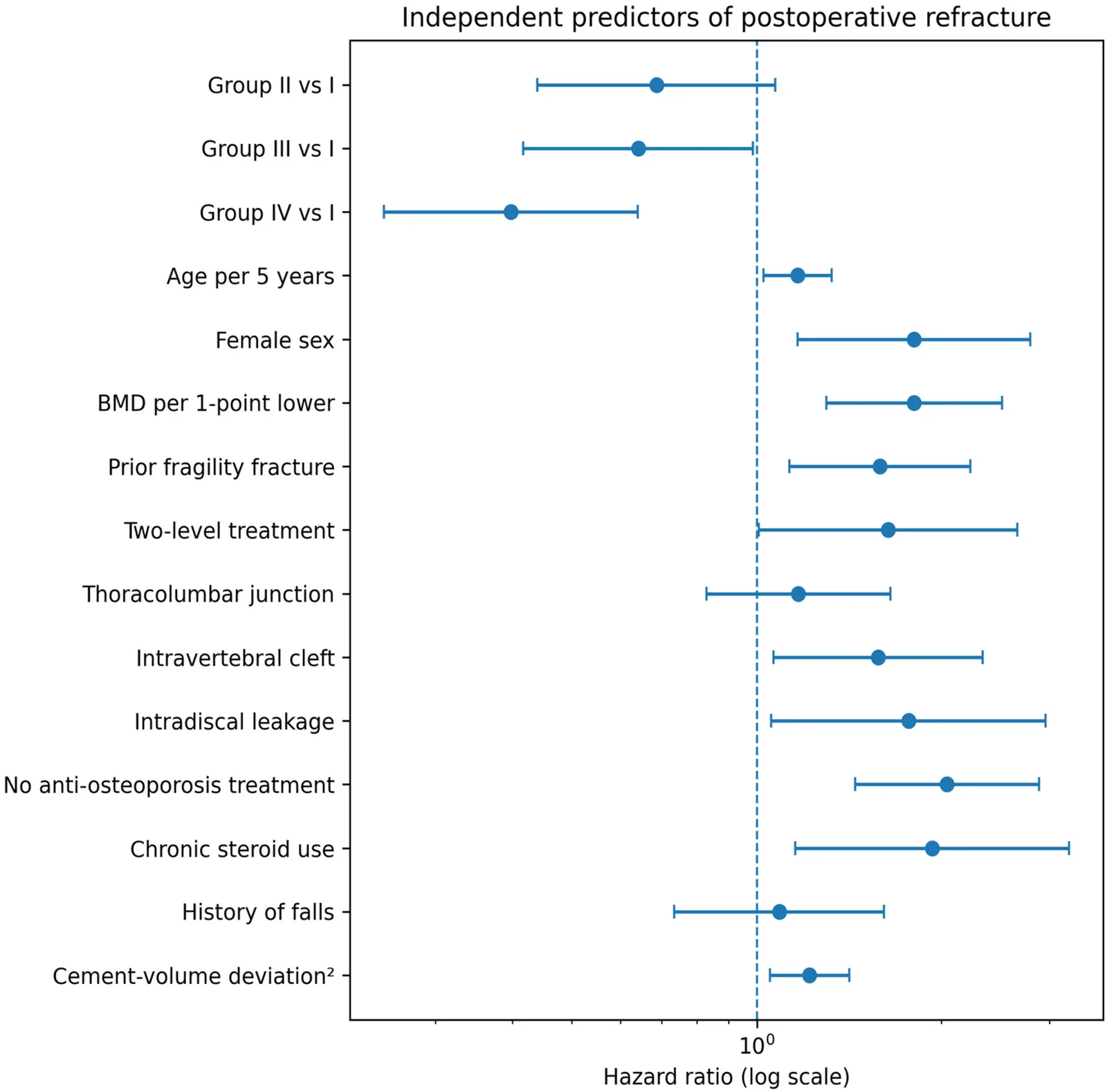
Adjusted hrs for postoperative refracture. The horizontal axis is logarithmic.

**Figure 9:**
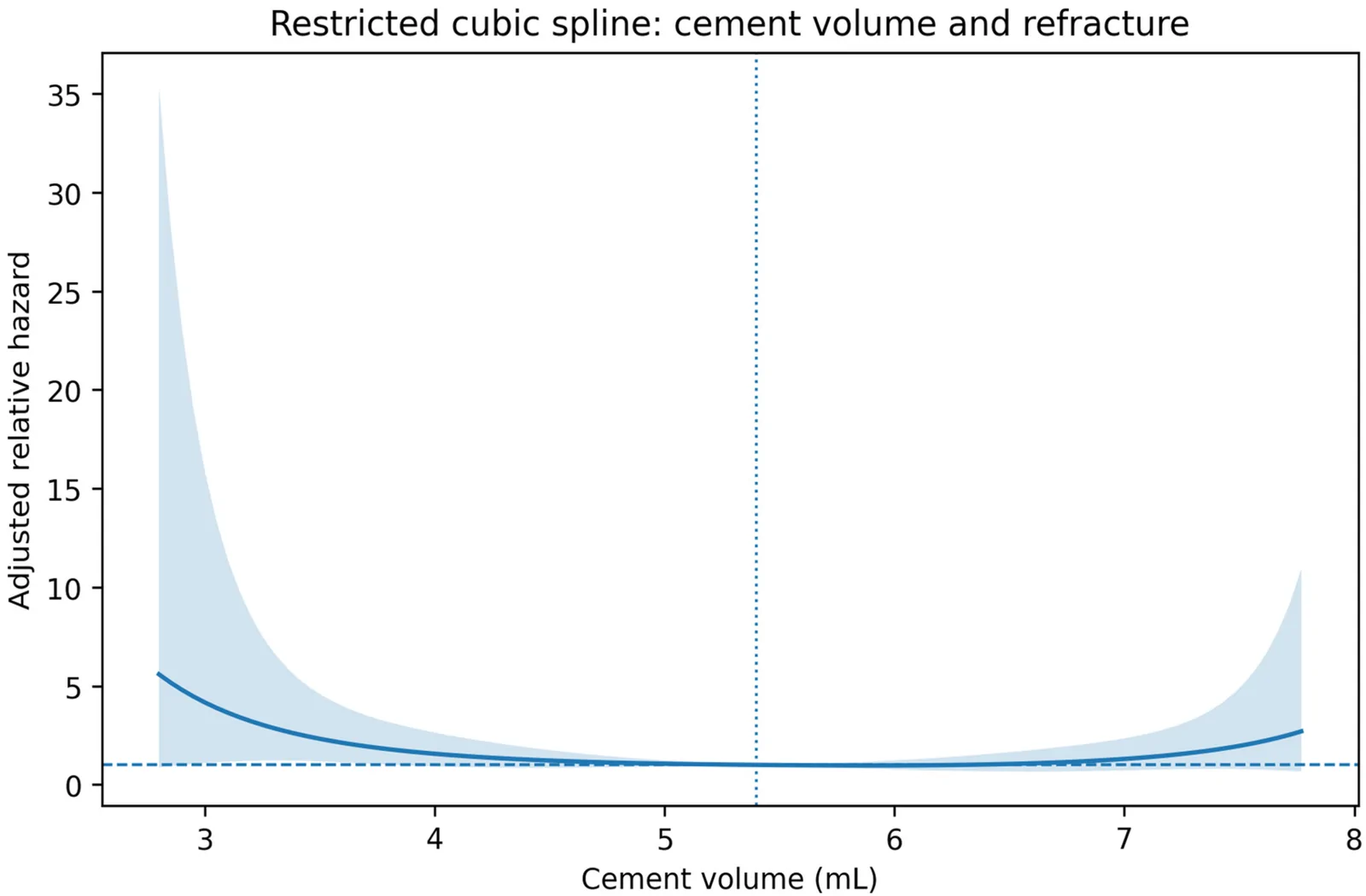
Restricted cubic spline for cement volume and modeled refracture risk.

**Figure 10:**
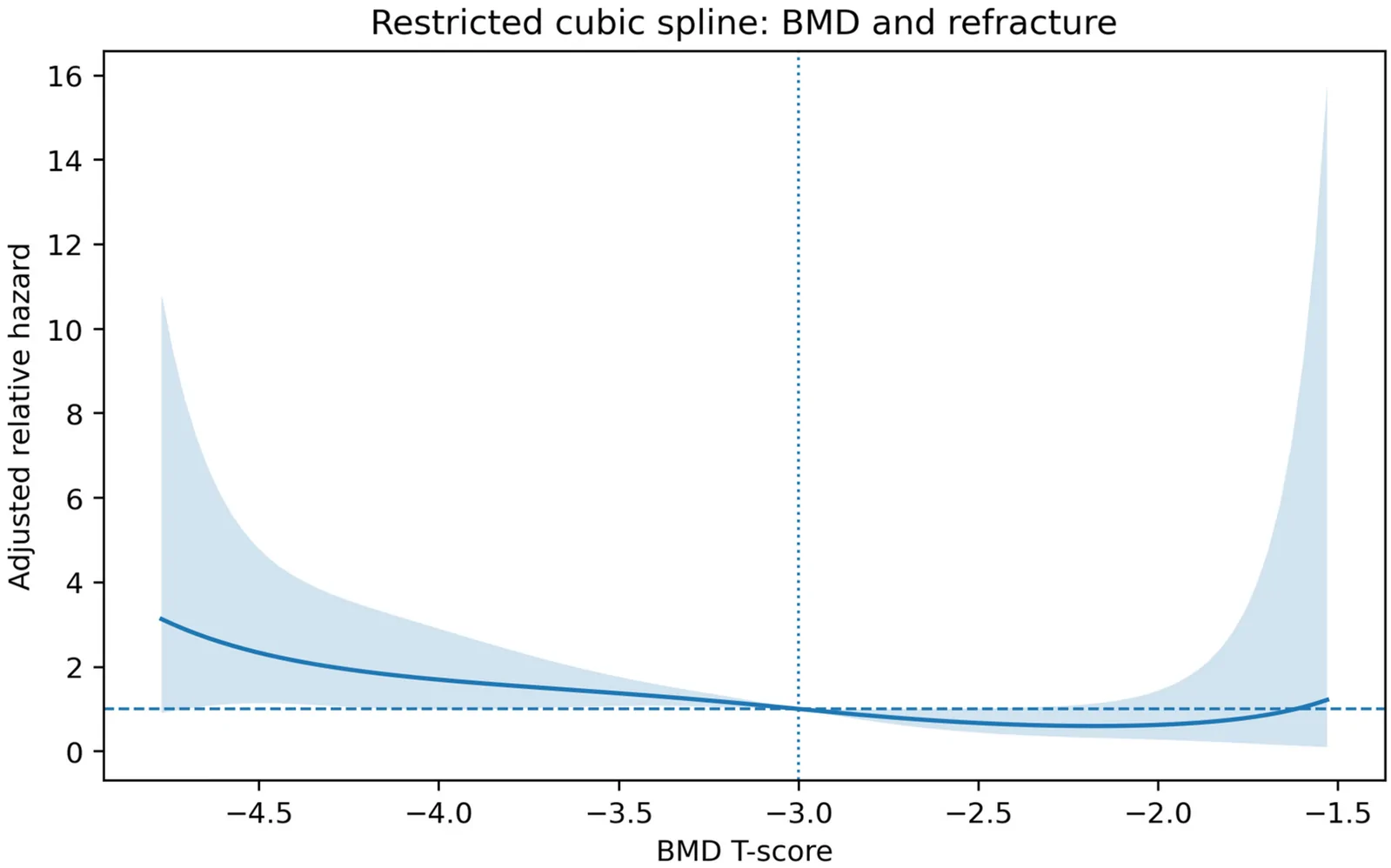
Restricted cubic spline for BMD T-score and modeled refracture risk.

Spline analyses supported a monotonic increase in risk as BMD declined and a U-shaped association between cement volume and refracture, with the lowest modeled risk near the midrange of the observed distribution. These findings should be interpreted as model-based associations rather than causal thresholds.

### 3.6 Sensitivity, subgroup, and prediction analyses

In the IPTW-weighted logistic regression, group IV had lower odds of 24-month refracture than group I, with an odds ratio (*OR*) of 0.41 (95% *CI* 0.24–0.68). After removing postoperative anti-osteoporosis treatment from the propensity-score model, the result was essentially unchanged (*OR* 0.42; 95% *CI* 0.25–0.69). In the volume-adjusted sensitivity analysis, group IV versus group I remained associated with a lower refracture hazard (*HR* 0.48; 95% *CI* 0.30–0.77; *P* = 0.002). Volume-stratified estimates were consistent: <5.0 mL, *HR* 0.52 (95% *CI* 0.29–0.93); 5.0–6.0 mL, *HR* 0.45 (95% *CI* 0.26–0.78); >6.0 mL, *HR* 0.55 (95% *CI* 0.28–1.08). The group × volume interaction was not significant (*P* = 0.41). In the sensitivity analysis including the 10 patients lost to follow-up before 24 months without an event, the revised cohort comprised 630 patients. The group IV versus group I association remained essentially unchanged (*HR* 0.41; 95% *CI* 0.26–0.65; *P* < 0.001), and the refracture rate was 23.8% (150/630) versus 24.2% (150/620) in the original analysis. These findings suggest that excluding these 10 patients did not materially bias the refracture estimates. When exposure was simplified to both-endplate contact versus any other pattern, the adjusted *HR* was 0.52 (95% *CI*, 0.34–0.79) in the full cohort and 0.53 (95% *CI*, 0.34–0.83) after restricting to single-level procedures. The piecewise complementary log-log model suggested a stronger association during months 0–12 (*HR*, 0.25; 95% *CI,* 0.11–0.57) than during months 13–24 (*HR*, 0.53; 95% *CI*, 0.19–1.50). Subtype analyses showed that the association was most evident for adjacent vertebral fracture, with a smaller and less precise association for index-level recompression and non-adjacent fracture See Figures 11–13 for details.

**Figure 11:**
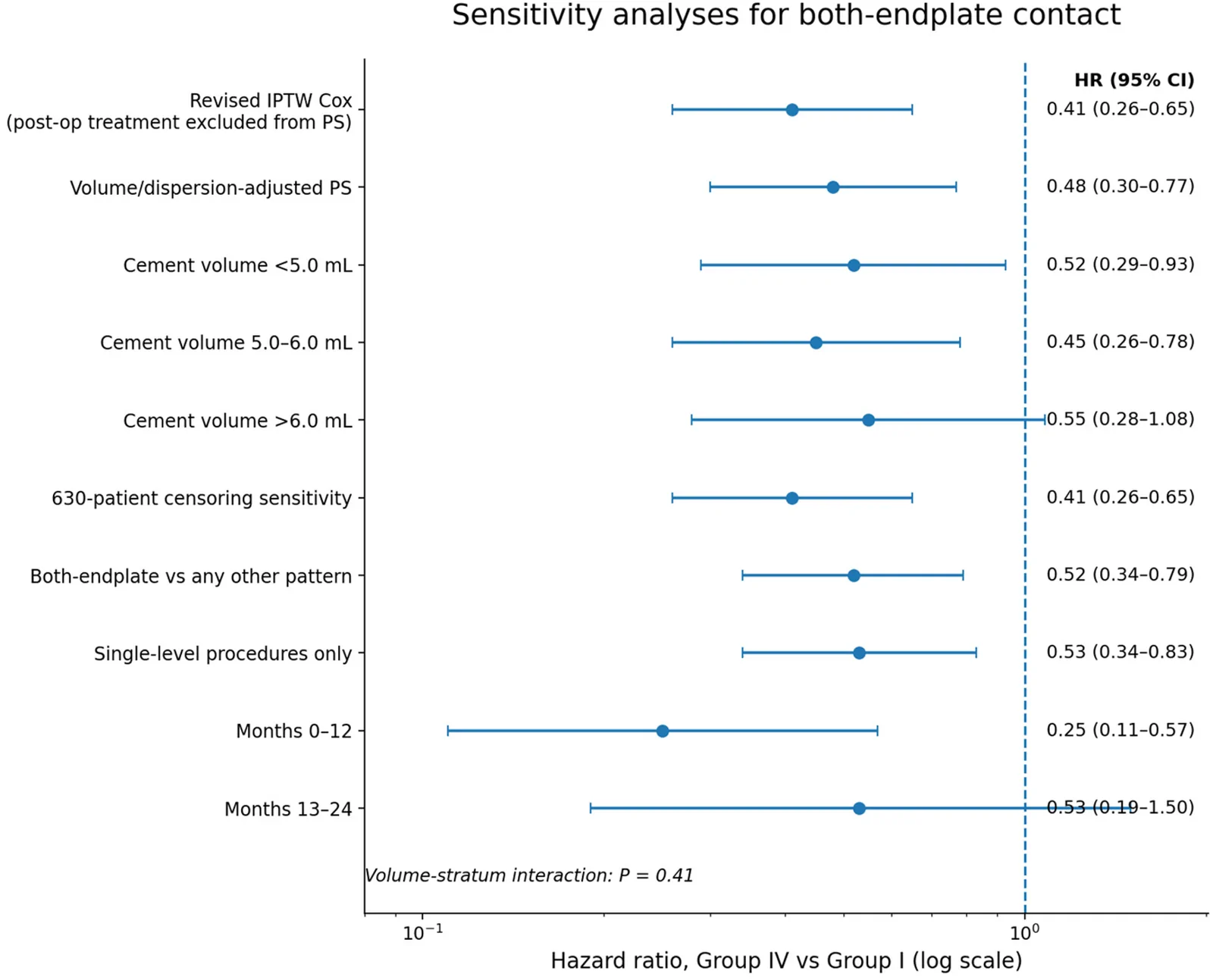
Sensitivity analyses for group IV versus group I. Points show HRs and horizontal lines show 95% CIs; the vertical dashed line denotes HR = 1.0. The group × volume interaction was *P* = 0.41.

**Figure 12:**
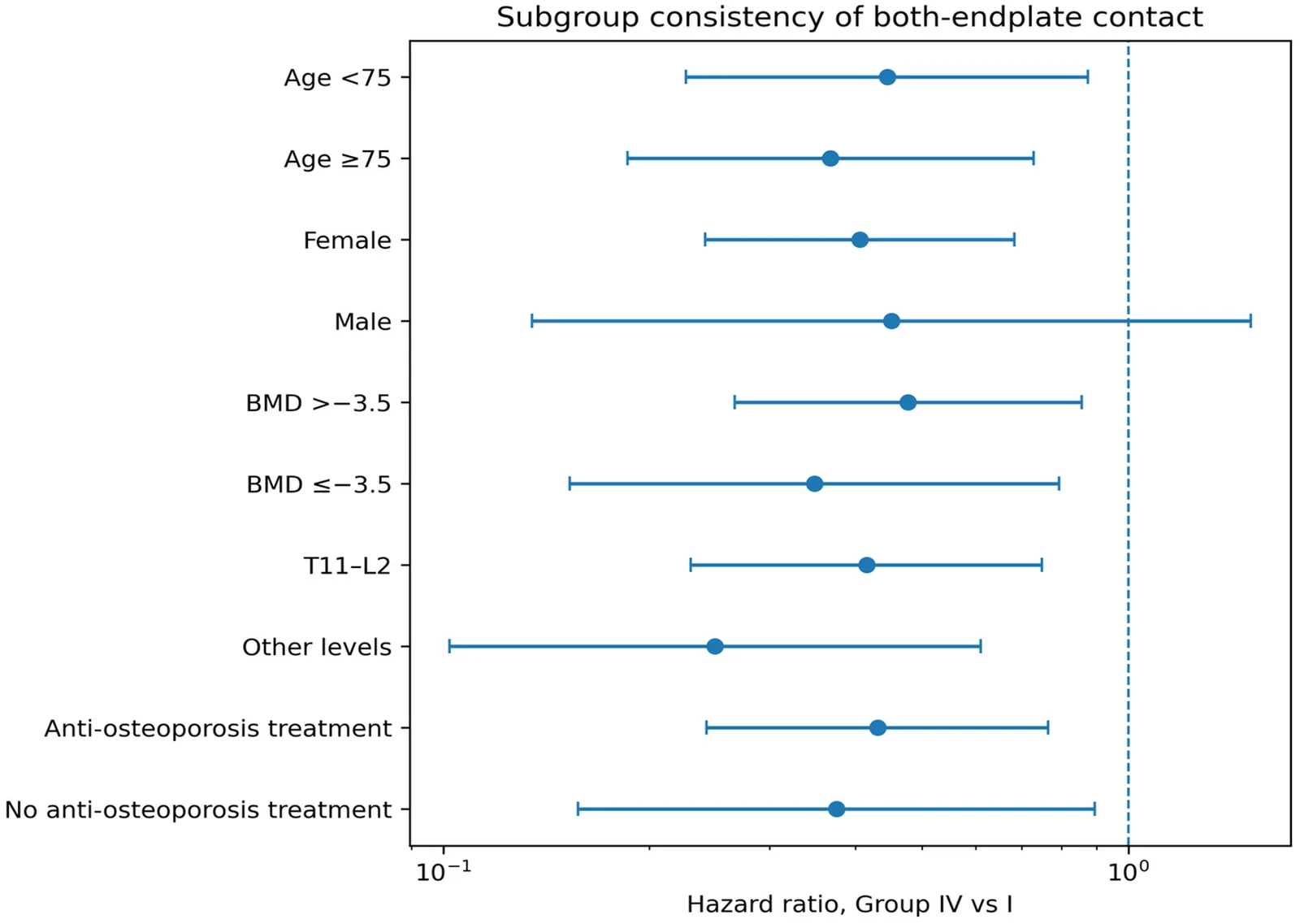
Prespecified subgroup analyses for group IV versus group I. Subgroup estimates are exploratory and not multiplicity-adjusted.

**Figure 13:**
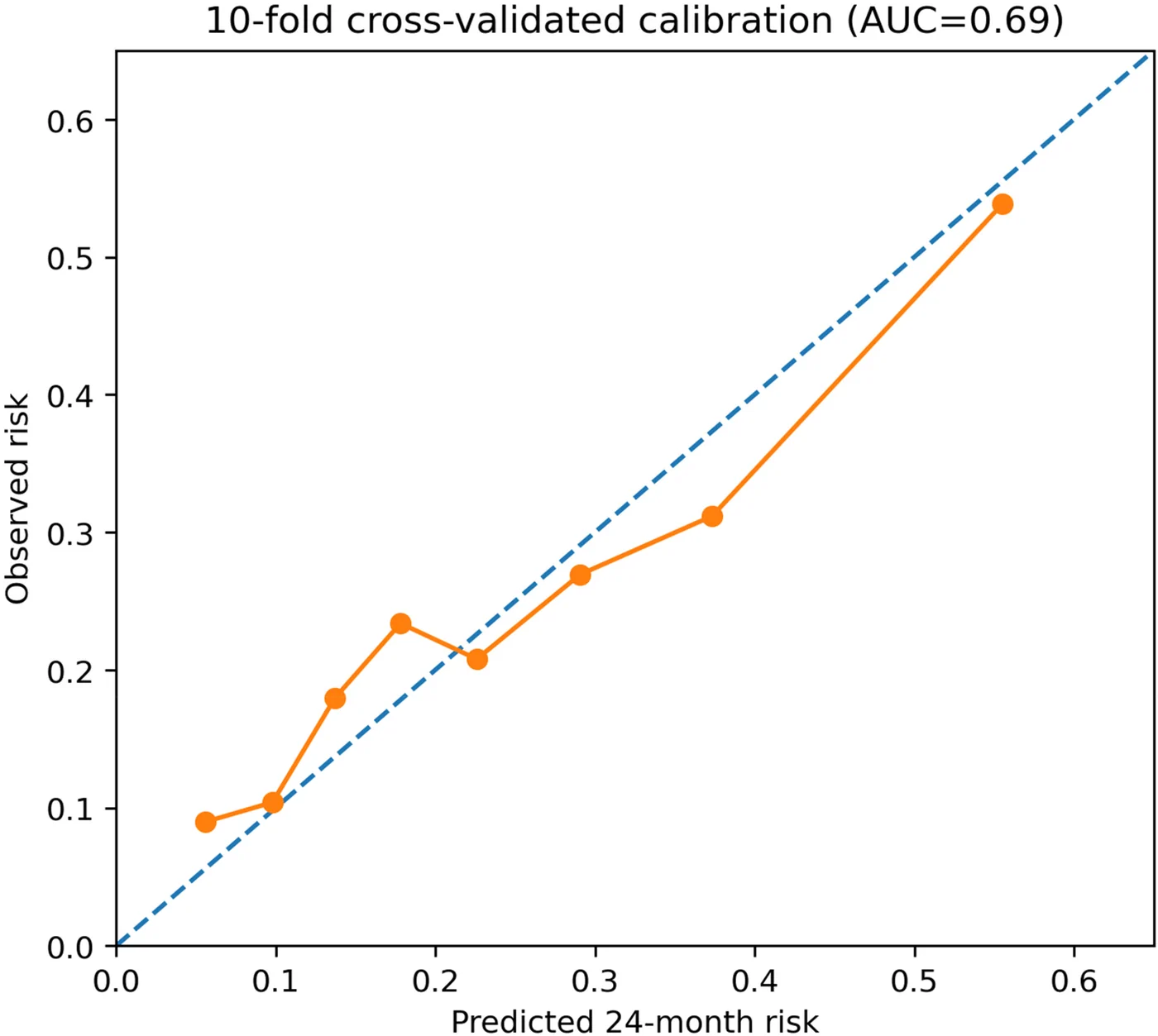
Cross-validated calibration of the exploratory refracture prediction model.

Subgroup estimates generally favored group IV, with wider *CIs* in smaller strata, particularly men. There was no convincing qualitative interaction by age, BMD category, fracture level, or anti-osteoporosis treatment. The exploratory 10-fold cross-validated prediction model yielded an *AUC* of 0.693 and a *Brier* score of 0.166, indicating moderate discrimination and reasonable average probabilistic accuracy in this deliberately heterogeneous cohort dataset.

## 4 Discussion

### 4.1 Principal findings

This analysis yielded four principal findings. First, cement contact with both endplates was associated with a lower refracture hazard after adjustment for demographic, skeletal, anatomical, procedural, and treatment-related factors. Second, group IV showed more durable improvement in pain and disability, with differences becoming more apparent during later follow-up. Third, postoperative refracture was independently associated with systemic skeletal fragility and procedural features, including low BMD, prior fragility fracture, glucocorticoid exposure, intradiscal leakage, two-level treatment, and absence of anti-osteoporosis therapy. Fourth, the relationship between cement volume and refracture was nonlinear, supporting a midrange rather than a maximal-volume strategy.

### 4.2 Cement distribution and mechanical interpretation

The direction of the observed association is consistent with clinical studies suggesting that broader cement dispersion and endplate support may create a more continuous load path and reduce unsupported cancellous regions (11, 12, 15, 16). The apparent tension between “double-endplate contact may require a larger volume” and “opposing volume maximization” can be resolved by distinguishing spatial distribution from total dose. Endplate-to-endplate contact is a spatial distribution pattern reflecting whether cement bridges and supports both endplates, whereas cement volume is a dose variable. Reaching both endplates may require a sufficient volume to bridge the fracture zone and contact both endplates, but “sufficient” is not equivalent to “maximal.” Beyond an optimal midrange, greater volume may increase leakage, stiffness mismatch, and adjacent-level stress. Consistently, the relationship between cement volume and refracture was U-shaped, supporting a midrange rather than maximal-volume strategy. In our cohort, group IV had a modestly higher mean cement volume than group I (5.75 vs. 5.31 mL; *Cohen's d* = 0.60), confirming that volume and endplate contact are correlated. However, after additional adjustment for cement volume and dispersion in the propensity-score model, the group IV versus group I association remained (*HR* 0.48; 95% *CI* 0.30–0.77; *P* = 0.002), and the group × volume interaction was not significant (*P* = 0.41). These findings suggest that the benefit of dual-endplate contact is not solely explained by greater cement volume. Therefore, the two concepts coexist: a sufficient volume may be needed to achieve dual-endplate support, but indiscriminate volume maximization is not advocated because excessive filling may increase leakage, stiffness mismatch, and adjacent-level stress. Finite-element analyses indicate that cement-endplate spacing changes stress transfer within the treated vertebra (17), while earlier radiographic work linked cement morphology with subsequent compression fracture (18). This trade-off argues against indiscriminate maximization of cement volume. A favorable construct is more likely to depend on controlled interdigitation, broad but non-extravasated dispersion, and adequate support of the fracture zone rather than a large compact cement mass.

The longer operative time and greater dispersion observed in group IV are also clinically plausible. Achieving endplate-to-endplate contact may require careful needle adjustment, staged injection, increased fluoroscopic vigilance, or advanced access techniques. Evaluations of dual-plane and curved unilateral trajectories indicate that altered delivery can improve cement distribution, although improved distribution does not invariably produce large early differences in pain (13, 14). Consistent with this pattern, early clinical separation was modest, whereas later differences were larger.

### 4.3 Refracture is a systems problem, not a cement-only problem

Older age, female sex, low BMD, prior fragility fracture, multiple treated levels, intradiscal leakage, glucocorticoid exposure, and absence of anti-osteoporosis treatment were independently associated with refracture. These variables overlap with recent systematic reviews (19–21) and cohort or biomechanical studies emphasizing adjacent-level risk, bone density, regional heterogeneity, and cement-related factors (22–25). A technically favorable cement pattern therefore cannot eliminate the systemic skeletal fragility that produced the index fracture. The subtype analyses further support the view that refracture is not a single mechanistic entity. Index-level recompression is more likely to reflect local mechanical support and cement distribution within the treated vertebra; adjacent vertebral fracture is more likely to reflect stress transfer, adjacent bone density, and cement stiffness; non-adjacent fracture is more likely to reflect systemic bone fragility, falls, and incomplete secondary prevention. The observed association was most evident for adjacent vertebral fracture, with smaller and less precise estimates for index-level recompression and non-adjacent fracture. These findings should be interpreted as exploratory because non-adjacent events were few.

The association of no anti-osteoporosis treatment with an approximately twofold hazard increase underscores the need to integrate augmentation into a fracture-liaison pathway. Contemporary evidence supports pharmacologic secondary prevention after vertebral fracture, with anabolic and antiresorptive options selected according to fracture risk, renal function, previous therapy, and contraindications (26). In real practice, the surgical encounter may be the best opportunity to initiate calcium and vitamin D assessment, evaluate secondary causes of osteoporosis, prescribe or arrange bone-active therapy, and implement fall prevention.

### 4.4 Cement volume, leakage, and nonlinear risk

The model used a quadratic cement-volume term because both insufficient and excessive filling can be problematic. Too little cement may fail to stabilize the fracture or bridge critical trabecular regions, whereas excessive cement may increase extravasation and stiffness mismatch. The observed *U-shaped* spline illustrates how categorizing volume into arbitrary “high” and “low” groups can lose information. In a real dataset, restricted cubic splines should be paired with careful inspection of the data range and *CIs*, and any apparent optimum should be externally validated rather than treated as a universal dosing target.

Intradiscal leakage, rather than overall leakage, was independently associated with refracture. This distinction is biologically plausible because disc contamination may alter endplate-disc mechanics and can be a marker of endplate disruption. Updated evidence syntheses and adjacent-fracture studies identify leakage, particularly disc leakage, among recurrent predictors of new or adjacent vertebral fracture (19–22). Nevertheless, imaging detection, leakage classification, and event adjudication vary across studies, so standardized radiological definitions are essential. The subtype analyses also suggest that adjacent vertebral fracture may be particularly sensitive to local mechanical and cement-related factors, whereas non-adjacent fracture may be more closely related to systemic skeletal fragility. However, these mechanistic interpretations remain hypothesis-generating.

### 4.5 Interpretation of VAS and ODI trajectories

All groups showed marked early improvement, consistent with the immediate stabilization and nociceptor-related mechanisms attributed to vertebral augmentation. The later divergence in ODI exceeded the early difference, suggesting that structural durability may influence cumulative mobility, sleep, lifting, and social function even when early pain relief is similar. Residual or recurrent pain after PVP is heterogeneous and may reflect recompression, a new fracture, facet or paraspinal sources, sagittal imbalance, or incomplete osteoporosis management (27–31). Longitudinal modeling is therefore preferable to isolated cross-sectional comparisons because it uses within-record information and evaluates whether group effects change over time. Although the adjusted VAS and ODI differences were statistically significant, their magnitude should be interpreted cautiously. The 24-month differences (VAS −0.72; ODI −6.75) were below commonly accepted MCID thresholds (VAS 1.5–2.0; ODI 10–15). Thus, the findings suggest a possible structural durability advantage but do not establish a clearly clinically important long-term functional benefit.

### 4.6 Clinical and research implications

For clinical research, the analysis demonstrates a pragmatic hierarchy. First, define cement distribution using a reproducible imaging rule and assess inter-rater reliability. Second, collect confounders that influence both distribution and outcome, including intravertebral cleft, fracture age, puncture route, cement viscosity, surgeon experience, and leakage. Third, analyze repeated VAS/ODI with a longitudinal model rather than repeated independent tests. Fourth, model refracture as time-to-event with clear censoring and competing-risk considerations. Fifth, report nonlinear relationships and temporal violations rather than forcing simple linear or proportional assumptions.

For future validation, a prospective multicenter registry should include postoperative thin-slice CT or standardized biplanar radiographs, blinded cement-pattern adjudication, DXA and vertebral HU, bone-turnover markers, detailed drug exposure, fall risk, and centralized adjudication of index, adjacent, and distant refractures. Targeted-emulation methods could compare distribution strategies while accounting for time-varying treatment and informative follow-up. Ultimately, procedural hypotheses should be tested in pragmatic randomized or strategy-based trials, especially as contemporary guidance and multidisciplinary pathways refine the role of vertebral augmentation (28–30, 32).

### 4.7 Strengths and limitations

The principal strength of the study is its methodological integration: a sample exceeding 500 records, a four-category exposure, repeated patient-reported outcomes, time-to-event analysis, multinomial weighting, center stratification, nonlinear terms, time-varying effects, subgroup analyses, and internal validation. The analytical plan emphasized effect estimates and CIs rather than reliance on *P* values alone.

This study has several limitations. First, as a retrospective single-center study, it is subject to selection bias and unmeasured confounding despite the use of IPTW and multivariable adjustment. Second, 10 patients were lost to follow-up before 24 months without a documented event and were excluded from the original analysis. In a sensitivity analysis including these patients and censoring them at last contact, the results were essentially unchanged, suggesting that this exclusion did not materially bias the refracture estimates. Nevertheless, residual selection bias cannot be entirely excluded. Third, cement volume and endplate contact are correlated; although volume-adjusted sensitivity analyses were performed, residual confounding by procedural technique and surgeon decision-making cannot be excluded. Fourth, postoperative anti-osteoporosis treatment occurred after exposure and was therefore not included in the propensity-score model; it was examined as a postoperative treatment variable in outcome models and sensitivity analyses. Fifth, cement-endplate contact was assessed using postoperative radiographs and CT, but interobserver variability and the inherent limitations of two-dimensional imaging may have affected classification accuracy. Sixth, the sample size, while adequate for the primary analysis, limited the precision of subgroup estimates, particularly in male patients and those with less common fracture patterns. Seventh, the composite refracture endpoint combines index-level recompression, adjacent vertebral fracture, and non-adjacent fracture, which may have different mechanisms. Exploratory subtype analyses were therefore added, but non-adjacent events were few and these analyses should be considered hypothesis-generating. Eighth, the follow-up period was restricted to 24 months, which may be insufficient to capture very late refractures or long-term anti-osteoporosis treatment effects. Ninth, center-level practice variation could not be assessed due to the single-center design. Therefore, our findings require external validation in larger, prospective, multicenter cohorts.

## 5 Conclusions

Cement contact with both vertebral endplates was associated with better late pain and disability trajectories, less recompression, and a lower 24-month refracture hazard after advanced confounding adjustment. However, the adjusted 24-month VAS and ODI differences did not exceed commonly accepted MCID thresholds, so the clinical magnitude remains uncertain. Older age, female sex, lower BMD, prior fragility fracture, two-level treatment, intravertebral cleft, intradiscal leakage, chronic glucocorticoid exposure, absence of anti-osteoporosis therapy, and deviation from a midrange cement volume were independently associated with refracture. These findings require validation in prospectively collected patient cohorts before they can inform clinical practice.

## Data Availability

The raw data supporting the conclusions of this article will be made available by the authors, without undue reservation.
